# Diffusion Spectrum Imaging of Corticospinal Tracts in Idiopathic Normal Pressure Hydrocephalus

**DOI:** 10.3389/fneur.2021.636518

**Published:** 2021-02-25

**Authors:** Hong Zhang, Wen-Jie He, Li-Hong Liang, Han-Wen Zhang, Xie-Jun Zhang, Liang Zeng, Si-Ping Luo, Fan Lin, Yi Lei

**Affiliations:** ^1^Department of Radiology, Health Science Center, Shenzhen Second People's Hospital, The First Affiliated Hospital of Shenzhen University, Shenzhen, China; ^2^Shantou University Medical College, Shantou, China; ^3^Department of Neurosurgery, Health Science Center, Shenzhen Second People's Hospital, The First Affiliated Hospital of Shenzhen University, Shenzhen, China

**Keywords:** idiopathic normal pressure hydrocephalus, diffusion spectrum imaging, corticospinal tract, quantitative anisotropy, diffusion magnetic resonance image

## Abstract

**Purpose:** The purpose of this study was to measure the diffusion spectrum imaging (DSI) parameters of corticospinal tracts (CSTs) and evaluate diffusional changes in CSTs in patients with idiopathic normal pressure hydrocephalus (iNPH) by DSI.

**Methods:** Twenty-three iNPH patients and twenty-one healthy controls (HCs) were involved in this study. Brain DSI data for all participants were collected through the same MR scanning procedure. The diffusion parameters measured and analyzed included quantitative anisotropy (QA), the isotropic diffusion component (ISO), general fractional anisotropy (GFA), fractional anisotropy (FA), mean diffusivity (MD), axial diffusivity (AD), and radial diffusivity (RD) of corticospinal tracts.

**Results:** The QA and ISO values of corticospinal tracts in iNPH patients were significantly lower than those in HCs (P_LQA_ = 0.008, P_RQA_ = 0.016, P_LISO_ = 0.024, P_RISO_ = 0.016). The mean MD, AD, and RD values in iNPH patients were significantly higher than those in HCs (P_MD_ = 0.032, P_AD_ = 0.032, P_RD_ = 0.048,). No significant differences in GFA and FA values were noted between iNPH patients and HCs.

**Conclusion:** Decreased QA and ISO values of corticospinal tracts were found in iNPH patients. Quantitative CST evaluation using DSI may lead to information that can improve the present understanding of the disease mechanism.

## Introduction

Idiopathic normal pressure hydrocephalus (iNPH) is a clinical syndrome that occurs in elderly people (1). Although the etiology remains unclear, it is a syndrome characterized by progressive gait disorder, cognitive impairment, and urinary incontinence (2, 3). Patients present with ventricular enlargement detected by radiological assessment with a normal range of cerebrospinal fluid (CSF) pressure (4, 5). Surgical shunt surgery is considered an effective treatment for iNPH (6).

Gait disorders are the most common and most characteristic symptom of iNPH, the mechanism of which is widely explained as enlarged ventricles mechanically compressing the paraventricular nerve fibers, especially corticospinal tracts (CSTs) (7). Previous studies used diffusion tensor imaging (DTI) to investigate subcortical involvement in the brains of iNPH patients and attempted to demonstrate white matter integrity changes (8). However, DTI cannot be used to evaluate the crossing and twining fiber bundles because it depends on a Gaussian parameterization of diffusion (9). Recently, diffusion spectrum imaging (DSI), a new kind of diffusion magnetic resonance imaging (dMRI) technique, has been used to noninvasively detect the complex white matter tract architecture and structural changes of fiber tracts in the human brain (10). DSI has been used to study some mental disorders such as autism and schizophrenia and neurodegenerative diseases such as Alzheimer's disease and multiple sclerosis (11–14). DSI reconstructs fiber tracts with a much higher resolution than conventional DTI and has been proven to accurately display crossing, winding, interrupted and small fibers (15, 16). The general fractional anisotropy (GFA) value is the main quantitative parameter of DSI, representing the directional consistency of water molecule diffusion (17). Generalized q-sampling imaging (GQI) (18) is one of the common reconstruction models for DSI data that can be used to calculate the indexes of quantitative anisotropy (QA) and the isotropic diffusion component (ISO) in addition to GFA.

However, the application of DSI in iNPH patients has not been reported at present. Thus, the purpose of this study was to evaluate diffusional changes in the corticospinal tract (CST) in patients with idiopathic normal pressure hydrocephalus by DSI.

## Materials and Methods

### Demographics and Clinical Data

**Figure 1:**
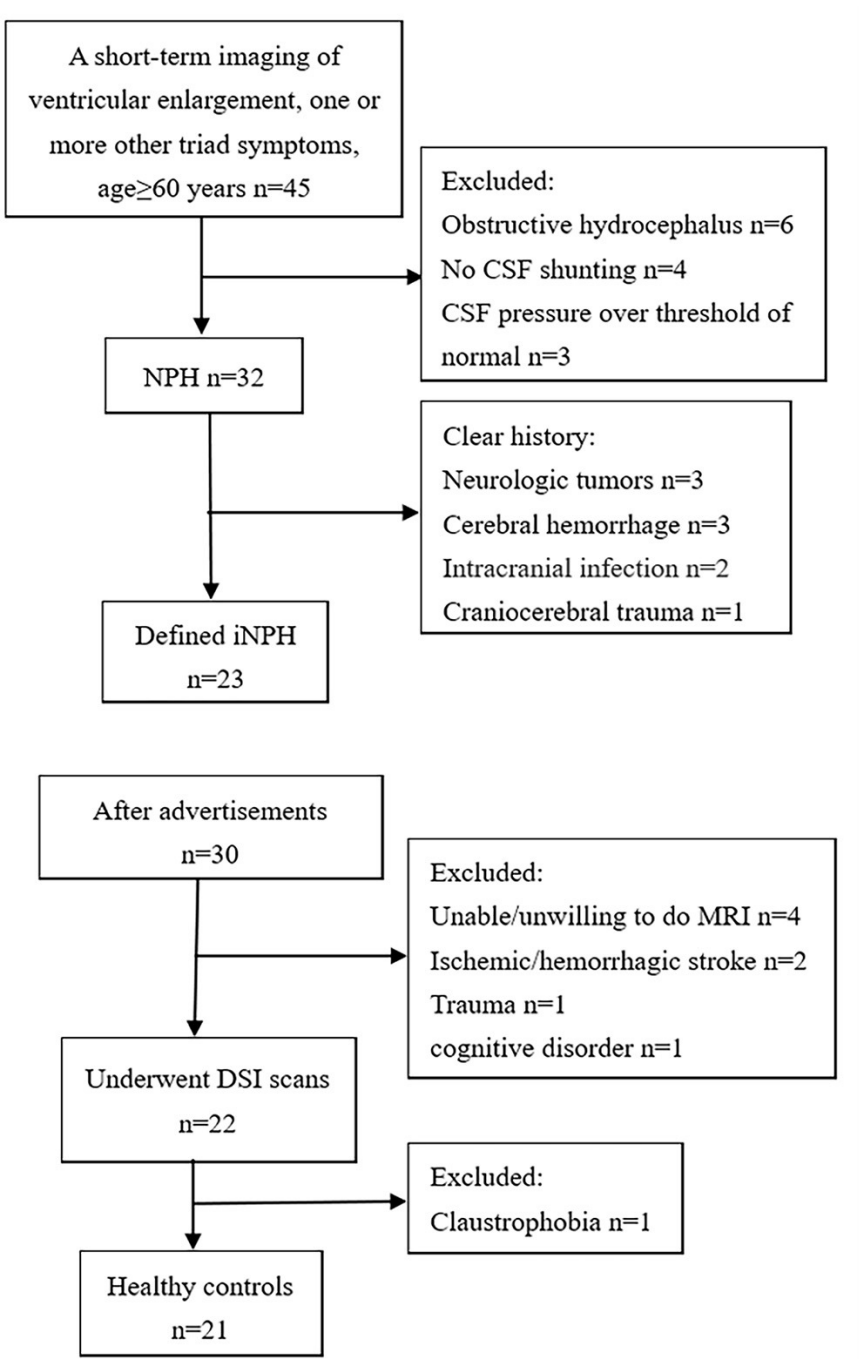
Flow chart of selection for the iNPH and HC groups in this study.

This study included 23 defined iNPH patients according to the guidelines for iNPH who were treated in the neurosurgery department of our hospital between March 2019 and May 2020 (1). Figure 1 shows the flow chart of the selection process for the iNPH and HC groups. Forty-two patients were included based on the following criteria: older than 60 years of age, presenting with one or more other typical triad symptoms (gait disturbance, cognitive disorder, and urinary incontinence), and the presence of a short-term radiology imaging finding indicating ventricular enlargement (19). Thirteen of these patients were excluded due to obstructive hydrocephalus (*n* = 6), no CSF shunting (*n* = 4), and abnormal CSF pressure (*n* = 3). Six patients with a clear history of neurologic tumors (*n* = 3), cerebral hemorrhage (*n* = 3), intracranial inflection (*n* = 2), and craniocerebral trauma (*n* = 1) that causes structural and functional changes in the brain were excluded. All recruited patients underwent the same MRI protocol and clinical assessments before CSF shunting.

The group of healthy controls (HCs) was recruited by placing advertisements in several health management centers and asking elderly individuals (over 60 years old) without a definite history of serious diseases such as ischemic or hemorrhagic stroke, trauma, or cognitive disorder to participate in this study. After history-taking and reading their imaging, 21 age- and sex-matched healthy volunteers were recruited as the HC group. All of the participants received the same brain MRI scans as the iNPH group. Age was included as the covariate in the statistical analysis because it was sensitive to the diffusion MRI indexes. Informed consent documents were signed prior to the examination by both groups of subjects. The protocol was approved by the Hospital Bioethics Committee.

### MR Imaging Acquisition

All subjects underwent MRI examination on a 3-Tesla MR scanner (Prisma; Siemens; Erlangen, Germany) with 20-channel phase-array head coils. The iNPH group was scanned before lumbar drainage or ventricular shunt surgery. The participants' heads were to remain stationary during the scanning as much as possible or with the help of a fixator if necessary. Data were collected using MRI sequences including T1-weighted magnetization-prepared rapid acquisition gradient-echo (MPRAGE) for better anatomic reference and diffusion spectrum imaging (DSI) using pulsed gradient twice-refocused spin-echo echo-planar imaging (EPI) sequences. T1-weighted sagittal images were obtained with 3D fast-field echo imaging. The following acquisition parameters were employed: repetition time, 2,300 ms; echo time, 3.55 ms; flip angle, 8°; slice thickness, 0.9 mm; number of sections, 192; field of view (FOV), 240 × 240 mm; matrix, 256 × 256; and voxel size, 0.9 mm × 0.9 mm. The acquisition parameters for DSI were as follows: repetition time, 6,300 ms; echo time, 71 ms; slice thickness, 2.2 mm; number of sections in the transverse plane, 60; field of view (FOV), 220 × 100 mm; matrix, 220 × 100; voxel size, 2.2 mm × 2.2 mm; and total diffusion sampling, 128, with a maximum diffusion sensitivity (*b*-values max) of 3,000 s/mm^2^.

### Tract Analysis

To analyze the microstructural integrity of the white matter fiber tracts, we used the DSI studio software (http://dsi-studio.labsolver.org/) to reconstruct the corticospinal tract (CST) of all participants. The diffusion MR images were imported in the DSI studio software, setting up the brain mask and using the generalized q-sampling imaging (GQI) reconstruction model (18) with a diffusion sampling length ratio of 1.25. Restricted diffusion was quantified using restricted diffusion imaging (20). CSTs were generated by manually placing seeds in the precentral motor cortex and placing targets in the cerebral peduncles (21). The volume sizes of the seeds were 2.8 × 10^3^ mm^3^ (left) and 3.0 × 10^3^ mm^3^ (right). The volume sizes of the targets were 1.3 × 10^3^ mm^3^ (left) and 1.4 × 10^3^ mm^3^ (right). These seeds and targets were adjusted to the precentral motor cortex and cerebral peduncles of each person to kept constant in all participants. A deterministic fiber tracking algorithm was used to reconstruct the CSTs (22). The angular threshold was 75 degrees. The step size was 1 mm. Tracks with lengths shorter than 20 or longer than 300 mm were discarded. A total of 5,000 tracts were calculated. Finally, the mean general fractional anisotropy (GFA), quantitative anisotropy (QA), normalized quantitative anisotropy (NQA), and isotropic diffusion component (ISO) values were calculated, as well as the elementary DTI parameters including fractional anisotropy (FA), mean diffusivity (MD), axial diffusivity (AD), and radial diffusivity (RD). The parameters of the right and left hemispheres were averaged for analysis if they had no significant difference and were analyzed separately if they had significant differences (21). Two radiologists with more than 10 years of experience in neuroradiology traced the seeds and targets separately and then evaluated the intergroup correlation coefficient of DSI parameters between the two tracers.

### Statistical Analysis

All statistical analyses were performed using SPSS version 25.0 software. One-way ANCOVA was used to compare the differences in age and sex between the iNPH patients and HCs. The Wilcoxon matched-pairs signed-ranks test was performed to test the differences in DSI-relevant values in the two hemispheres. The Mann–Whitney *U-*test was used to compare the differences in DSI values between the iNPH and healthy control groups. Bonferroni correction was used to correct the multiple comparisons of different groups. The adjusted statistical significance level was accepted at 0.05 (two-tailed).

## Results

In total, 23 defined iNPH patients (age 70.2 ± 9.1 years; range 60–81 years; 13 men and 10 women) and 21 healthy volunteers (age 69.7 ± 8.8 years; range 60–80 years; 11 men and 10 women) were included in this study. Sex and age were not significantly different between the two groups of subjects. The objective symptoms on the iNPH Grading Scales were gait disturbance in 100% of the patients, cognitive impairment in 91.3% of patients, urinary symptoms in 56.5% of patients, and the triad of symptoms in 56.5% of patients (Table 1). All of the patients presented with varying degrees of gait disturbance.

**Table 1:** Demographic data and clinical characteristic of the iNPH patients and HCs.

**Characteristics**	**iNPH patients (*n* = 23)**	**HC (*n* = 21)**	**P-value**
Gender, male	13 (56.5)	11 (52.4)	0.723
Age (year)	70.2 ± 9.1	69.7 ± 8.8	0.146
**Symptoms present**			
Triad of symptoms	13 (56.5)	N/A	
Gait + cognitive impairment	8 (34.8)	N/A	
Gait + urinary symptom	0	N/A	
Cognitive impairment + urinary symptom	0	N/A	
Gait only	2 (8.7)	N/A	
Cognitive impairment only	0	N/A	
Urinary symptom only	0	N/A	

Data are presented as number (%) of patients.

*N/A, Not Applicable*.

**Figure 2:**
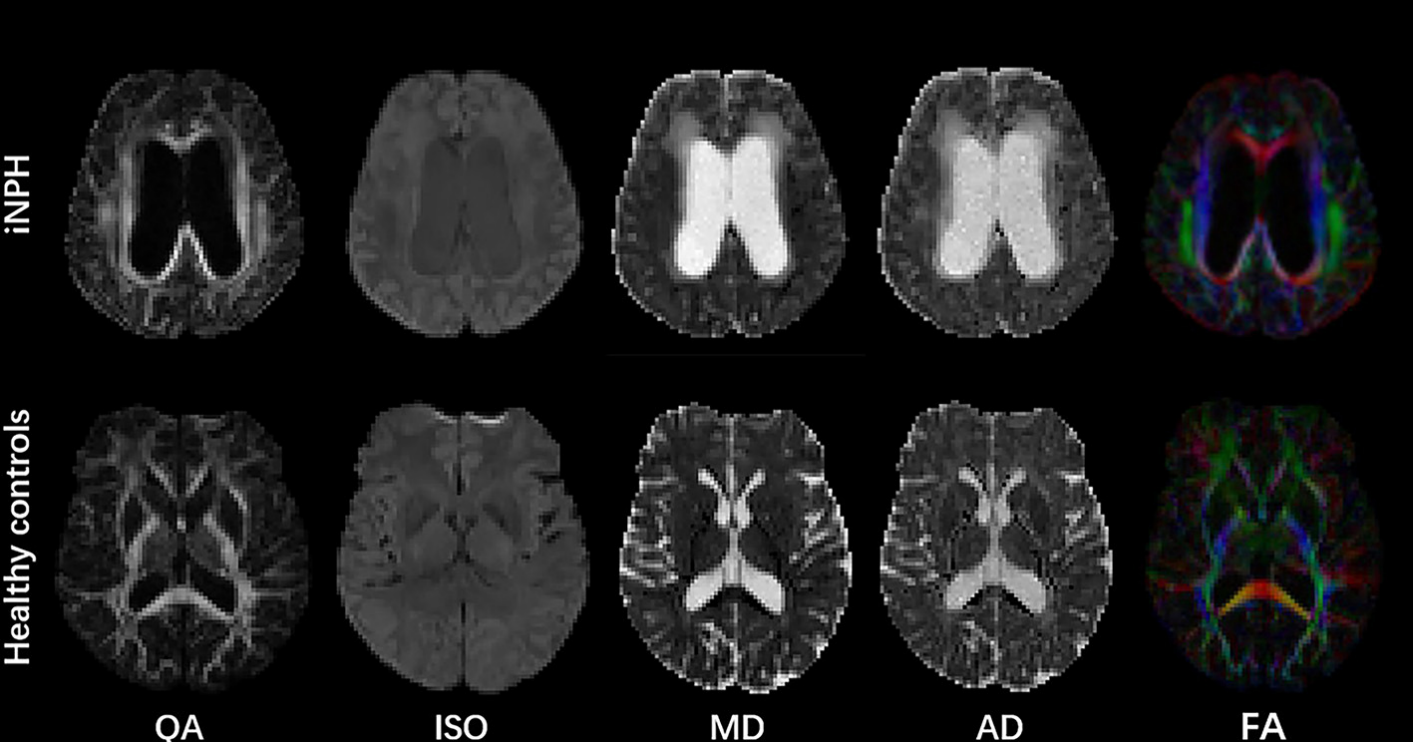
Parametric maps of QA, ISO, MD, AD, and color FA values of the iNPH patient and HC samples.

**Figure 3:**
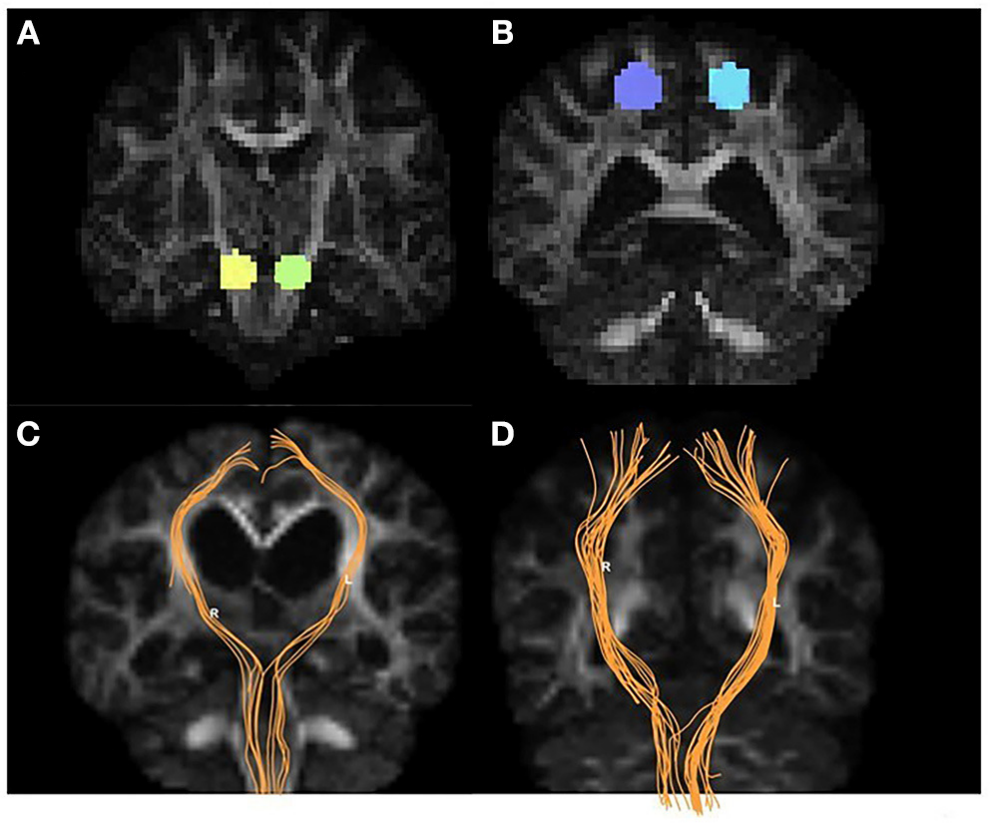
Examples of volumes of interest, CST tracking, and CST reconstruction. Panel **(A)** shows the targets placed on two sides of the cerebral peduncle (yellow and green). Panel **(B)** shows the seeds placed on the precentral gyrus (purple and blue). The CST tractography (orange) for iNPH patients **(C)** and HCs **(D)**.

Figure 2 shows the parametric maps of the QA, ISO, MD, AD, and color-FA values of the iNPH and HC samples. After tracking and reconstructing the corticospinal tracts of both hemispheres in each subject, the DSI parameters of the whole tracts were calculated separately. Two neuroradiologists measured and calculated the parameters separately, and the intergroup correlation coefficients (ICCs) of these measurements were ICC_QA_ = 0.92, ICC_ISO_ = 0.96, ICC_MD_ = 0.93, ICC_AD_ = 0.92, ICC_FA_ = 0.89, ICC_RD_ = 0.90, ICC_GFA_ = 0.92, and ICC_NQA_ = 0.90. The parameters were averaged and analyzed. Figure 3 shows the sample of corticospinal tracts reconstructed by deterministic tracking for patients with iNPH and healthy controls. The mean QA values and mean ISO values were not significantly different between the right hemisphere and left hemisphere (P_QA_ = 0.147, P_ISO_ = 0.159) in iNPH patients, while the HCs had significant differences between the two hemispheres (*P* = 0.006, 0.002, respectively). Other mean values of FA, MD, AD, RD, and GFA did not significantly differ between the two sides of hemispheres.

**Figure 4:**
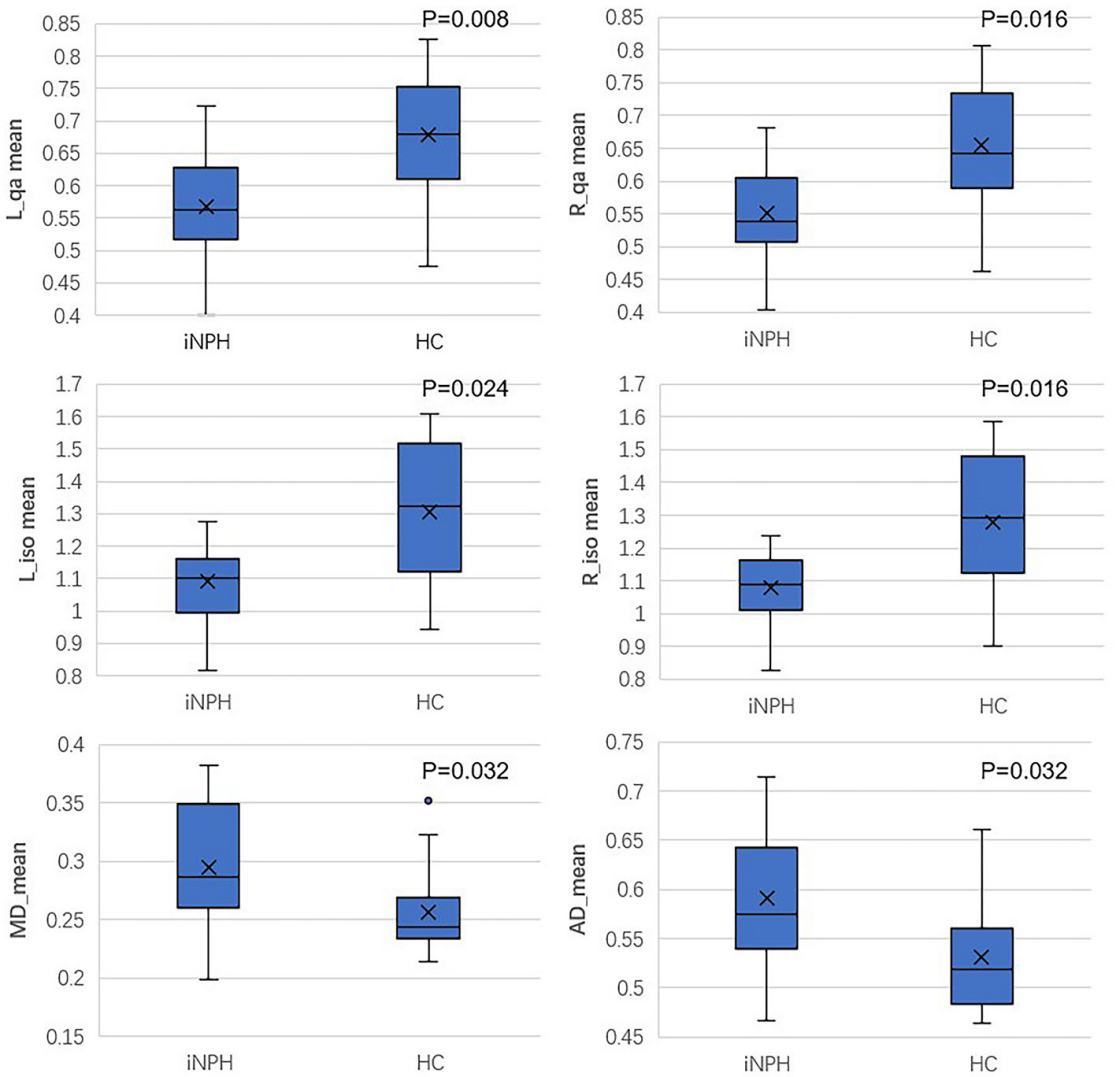
Boxplot of QA, ISO, MD, and AD values between iNPH patients and HCs.

Table 2 shows the different values measured from corticospinal tracts and the significant difference between iNPH patients and healthy controls. Compared with the healthy volunteers, the QA and ISO values of the two sides of the corticospinal tracts were both significantly reduced in iNPH patients (P_LQA_ = 0.008, P_RQA_ = 0.016, P_LISO_ = 0.024, and P_RISO_ = 0.016). The mean MD, AD, and RD values were all significantly different between iNPH patients and healthy controls. Mean MD, AD, and RD values in iNPH patients were increased compared with those of HCs. The differences in the QA, ISO, MD, and AD values of the two groups are presented in Figure 4. Regarding the mean FA and GFA values, there was no significant difference between iNPH patients and healthy people.

**Table 2:** Values measured in corticospinal tracts and P values of the iNPH patients and HCs.

	**iNPH (Mean)**		**HC (Mean)**		**Corrected** ***P-*****value**	
	**L**	**R**	**L**	**R**	**L**	**R**
QA	0.567	0.551	0.679	0.655	0.008^*^	0.016^*^
ISO	1.091	1.078	1.304	1.279	0.024^*^	0.016^*^
FA	0.749		0.781		0.200	
MD	0.295		0.256		0.032^*^	
AD	0.591		0.531		0.032^*^	
RD	0.147		0.119		0.048^*^	
GFA	0.109		0.109		1.000	

QA indicates quantitative anisotropy; ISO, isotropic diffusion component; FA, fractional anisotropy; MD, mean diffusivity; AD, axial diffusivity; RD, radial diffusivity; GFA, general fractional anisotropy.

* *P < 0.05*.

## Discussion

In this study, we explored the corticospinal tract DSI parameters in patients with iNPH. We found that among the DSI parameters, the QA values, and ISO values of the CSTs for both the left and right sides were significantly different from those of healthy controls. On the other hand, regarding the traditional DTI parameters, the MD, AD, and RD values showed significant differences between iNPH patients and the HC group, while the FA values showed no significant differences between them.

Our study shows that QA values of bilateral corticospinal tracts were decreased in patients with iNPH compared with the QA values of HCs. Meanwhile, we found that there was no significant difference in the GFA values of corticospinal tracts in iNPH patients. GFA and QA values both represent the degree of anisotropy of the fiber bundles in DSI parameters. Some studies have suggested GFA as the main quantitative indicator of DSI to use for analyses (11). However, several studies have demonstrated that GFA is easily sensitive to the partial volume effects of crossing fibers and edema, suggesting that it is not a stable index (22, 23). In fact, iNPH patients usually have periventricular white matter hyperintensities and periventricular edema. Thus, we suggest that GFA might not be a good parameter for evaluating changes in CSTs in iNPH patients. In contrast, the QA value is calculated from the reconstruction model of generalized q-sampling imaging (GQI) using DSI (24), which separates the ISO components of the fibers. Thus, QA is not susceptible to edema and crossing fibers but is more suitable for the analysis of hydrocephalus because it is always influenced by exudation. This allows QA values to reflect the changes in the fiber itself more accurately and reliably. A reduction in QA values reflects the damage to the integrity of the axon or myelin sheath in principle (18).

In addition, the ISO values represent the isotropic diffusion of water molecules within cells or between cells, which is specific in DSI analysis in principle. We found that the ISO of the patient group was significantly lower than that of the control group. Combining QA and ISO, we suggested that this was possibly because the intercellular space was smaller and the isotropic diffusion of water molecules was restricted, which was caused by the compression of the lateral ventricle enlargement.

Studies of directional diffusion parameters such as FA values were found to be elevated in the corticospinal tracts of patients with iNPH (8). The FA values also showed significant differences in the axons of white matter in iNPH patients compared with patients with neurodegenerative diseases and healthy groups in previous studies (25). Our study found that the MD and AD values increased in iNPH patients, which is consistent with the results in the literature that implied an increase in the diffusion of water molecules. The increased MD and AD values could be caused by periventricular edema in iNPH patients, in accordance with a previous study observing that periventricular edema can lead to increased diffusion (26). However, our results show that the FA values were not significantly different between patients with iNPH and HCs, which is different from the findings of previous studies. According to the previous perspective, the enlarged lateral ventricles mechanically compressed the axons of corticospinal tracts and made the water molecules align along the axon orientation, which caused the FA values to be elevated (27, 28). In fact, mechanical pressure may cause atrophy and irreversible damage of the axons (21), which make FA values decrease accordingly. Combined with the above DSI parameters and FA in principle, we hypothesized that the lack of difference in FA values in our study was due to the coexistence of compression and injury. Thus, the variation in FA values could be canceled out.

Gait disturbance is always the first and foremost symptom in iNPH patients and is considered to be related to the injury to corticospinal tracts (29). The whole corticospinal tract is composed of complex fibers and intersects with many other fibers. We compared the diffusion parameters in CSTs of iNPH patients with those of age-matched healthy individuals. Our results show that the QA was significantly decreased in the patient group, but the most commonly used DTI parameter FA presented no significant difference between the two groups. The DTI model is unable to address such complex areas with various fiber directions, so it has some limitations in the analysis of white matter (30). DSI counterbalances the deficiency of DTI and has the ability to reveal crossing fibers, which can be used to show the complex neural network connectivity and microstructure of the brain (31, 32). DSI indicators were able to reflect the microscopic changes in the fiber structure more accurately. Our studies also found a difference in QA in the two hemispheres of CSTs in normal people. We suggest that this difference might be influenced by the difference between the dominant hemispheres. Nevertheless, these discrepancies were not observed in DTI indicators. Although there are other diffusion imaging studies in iNPH, this is the first DSI study providing a new diffusion method and complementary parameters for investigating alterations in nerve fibers in iNPH patients, enabling to widen the DTI findings.

This study also has some limitations. The relatively small sample size from a single center might limit the intergroup differences of some parameters, thereby preventing statistical significance from being achieved to a certain extent. This is a preliminary study, and longitudinal research on patients with iNPH in the future will be helpful in the preoperative evaluation of patient outcomes and can verify the accuracy of this study. Future research endeavors should be expanded to include more elderly people with neurodegenerative diseases who have some clinical manifestations similar to iNPH.

## Conclusion

We found that iNPH patients present with decreased quantitative anisotropy, decreased isotropic diffusion component and increased mean diffusivity, radial diffusivity, and axial diffusivity in their corticospinal tracts. This reflected not only the compression of fibers by enlarged ventricles but also the change in the integrity of microstructural fibers, which helped us to better understand the mechanism of the disease.

## Data Availability Statement

The raw data supporting the conclusions of this article will be made available by the authors, without undue reservation.

## Ethics Statement

The studies involving human participants were reviewed and approved by Research Ethics Committee of the First Affiliated Hospital of Shenzhen University. The patients/participants provided their written informed consent to participate in this study.

## Author Contributions

HZ, W-JH, FL, and YL: guarantors of integrity of entire study and manuscript editing. HZ and W-JH: study conception/design, data analysis/interpretation and manuscript drafting, or revision for important intellectual content. HZ, W-JH, H-WZ, and L-HL: literature research and data analysis. HZ, S-PL, X-JZ, and LZ: data acquisition or clinical studies. All authors: approval of final version of submitted manuscript and agrees to ensure any questions related to the work are appropriately resolved.

## Conflict of Interest

The authors declare that the research was conducted in the absence of any commercial or financial relationships that could be construed as a potential conflict of interest.

## Abbreviations

P_LQA_: *p* value for QA metrics of the left CST
P_RQA_: *p-*value for QA metrics of the right CST
P_LISO_: *p-*value for ISO metrics of the left CST
P_RISO_: *p-*value for ISO metrics of the right CST
P_MD_: *p*-value for MD metrics
P_AD_: *p*-value for AD metrics
P_RD_: *p-*value for RD metrics.
